# Exploring critical factors of the perceived usefulness of blended learning for higher education students

**DOI:** 10.1371/journal.pone.0223767

**Published:** 2019-11-21

**Authors:** Damijana Keržič, Nina Tomaževič, Aleksander Aristovnik, Lan Umek

**Affiliations:** Faculty of Public Administration, University of Ljubljana, Ljubljana, Slovenia; National University Ireland Galway, IRELAND

## Abstract

Performed in a Slovenian higher education institution, the presented research was designed to help investigate which factors influence the ways a student perceives an e-course’s usefulness in a blended learning environment. The study is based on an online questionnaire completed by 539 students whose participation in the survey was voluntary. Using structural equation modelling, the students’ perceptions of different aspects were investigated, including their attitudes to course topics and technology, learning preferences, teachers’ role in course design and managing the teaching process. The empirical results show e-learning is positively perceived to be usefulness when: (1) the teacher is engaged and their activities in an e-course, with the (2) a student’s attitude to the subject matter and the lecturer’s classroom performance having a direct impact, and (3) technology acceptance having an indirect impact. No major differences were revealed when the model was tested on student subgroups sorted by gender, year of study, and students’ weekly spare-time activities.

## Introduction

In the last two decades, the growing role of information and communications technology (ICT) has been facilitated by online learning, also known as e-learning or web learning, where ICT is used to deliver information related to a course and gives access to study contents, assignments and learning tasks. While there is no exact definition of individual terms in the literature, some authors distinguish them yet others use them simultaneously [1]. For this article, e-learning means computer (or mobile media) access to a learning environment via the Internet, unlimited by time or place, typically not in the classroom. The rapid development of e-learning environments like learning management systems (LMS) that support and enable effective e-learning as part of the education process has seen many changes being made to the methodology and techniques of teaching and learning [2], with e-learning becoming ever more important in educational institutions and emerging as a new paradigm in modern education [3,4]. By combining traditional face-to-face classroom teaching (synchronous learning activities) with e-learning (asynchronous learning activities), blended learning (BL) is already commonly used in teaching and learning in higher education.

BL is seen as a promising teaching and learning principle in many higher education institutions. It achieves learning objectives by applying state-of-the-art technologies in order to customize the act of learning and transfer knowledge and skills to the right person at the right time and, combined with face-to-face learning, maintains regular social interactions among colleagues and teachers. Therefore, BL is a combination of “different delivery methods and styles of learning” [5]. The proportion between these two teaching-learning concepts may vary, but it is suggested [6] that the e-learning component ranges between 33% and 50%.

Yet, BL should not simply be seen as a substitute for face-to-face classroom and online instruction focused on time-based measures; crucially, we must consider the progress being made by a student in the online environment (Piper, 2010, as cited [7]). BL can maximize the benefits of both face-to-face methods in classroom lectures and online education with asynchronous and/or synchronous online learning [5]. Compared with face-to-face instructions, online instructions rely on extensive use of LMS functions and functionalities, enable objectives to be efficiently specified, organize materials, and facilitate engagement and the assessment of outcomes [8], while making student–teacher interaction easier, more efficient, allowing personalization and significantly overcoming time and space limits [2]. Nevertheless, such a wide range of benefits does not mean pedagogical signals should not be carefully monitored [9]. Diep *et al*. [10] recently highlighted the “modalities” aspect, the degree to which BL is implemented in the pedagogical process, and concluded that in a more intensive BL environment LMS has a greater influence on a student’s satisfaction with e-learning.

This rapid expansion into all areas of education is encouraging new explorations of how e-learning impacts study results; what affects the dropout rate [11], which key factors help with a positive attitude and satisfaction in e-learning [12–14], when do students say an e-course is effective and useful for their studies [15–17], what affects the success rate of a student involved in an e-course [18], and which critical features of e-learning ensure efficient learning [19]. Research is still ongoing, while the many contradictory results emerging to date show the final word on the subject remains elusive [12].

To successfully employ BL in higher education, one must know how well e-learning supports the face-to-face lectures, if the prepared e-content is appropriate, or whether an e-course is merely an end in itself. BL requires complex teaching strategies where, in addition to supporting face-to-face lectures, the work, contributions and other activities of the students in an e-course require continuous monitoring. To sum up, in the last two decades the research has focused on various critical (success) factors of e-learning (as part of BL or fully online learning), placing them in three main groups:

1. student factors–prior experience/knowledge of IT [20], self-efficacy, self-motivation, learning style and responsibility for one’s own learning pace [18,21,22];
2. teacher factors–characteristics [23], ICT competencies [24], teaching style [25], knowledge, facilitation, feedback and course structure [22], online instruction [21], information quality and service delivery quality [15,18]; and
3. technology acceptance and technical support–ease of use, ease of access, user-friendly interface, technical support [15,21,26,27].

Knowing what grabs the attention of students and understanding the factors that influence students’ success in e-learning environment can help academic management and teachers create mechanisms to encourage students to adopt this learning environment. This makes it necessary to conduct research that more intensively considers learners’ perception of, attitude to, and intention to use e-learning [26]. A recent literature review [28] outlines four challenges of ‘how to’ design BL: integrating flexibility (autonomy of time and place, pace of study), encouraging interaction (interaction in face-to-face and online environments), assisting students’ learning process (activities regulated by teachers to help students–monitoring, evaluating), and fostering an effective learning climate (providing motivation and encouragement, showing empathy, attending to individuals). If the students feel the e-learning system is useful for the study, they are motivated to use it. Yet, it is evident that some students are reluctant to use LMS [29].

The study presented here intends to provide insights into the BL’s perceived usefulness in higher education from the end-users’ perspective, i.e. students. The research was conducted among students of the Faculty of Public Administration (FPA), a member of the University of Ljubljana. The FPA offers two undergraduate study programmes (1st cycle)–(1) University Study Programme in Public Sector Governance; and (2) Higher Education Professional Study Programme in Administration. Since the 2012/13 academic year, every undergraduate study course has had its own e-course in LMS Moodle whereby approximately 70% of a course entails traditional face-to-face class teaching and the remaining 30% of the pedagogical process an e-course, namely, at the lower end of the recommended range for the proportion of blending learning [30]. The e-course study materials support face-to-face lectures with supplementary readings followed by a self-assessment activity, usually a quiz or an assignment, to check the understanding of the online content. Further, three extensive assignments (instead of classroom tutorials) are prepared over a 15-week semester. The teacher reviews and gives feedback to the students about the correctness of the solutions they submit [31]. Such formative assessment gives students not only information about the acquired knowledge but also their learning. Conversely, the teacher gains insights into the students’ understanding, learning needs for further explanation, and is able to monitor their progress.

We formulated two research questions to be discussed in this paper:

1. Which factors influence an e-course’s perceived usefulness while studying for the final exam?
2. Are the most significant impact factors the same when analysing subgroups of students determined by demographic characteristics, e.g. gender, year of study, university programme or regular weekly spare-time obligations?

### Proposed research model

In the study presented here, the focus was on a student’s subjective opinions on different aspects of BL in order to determine those factors influencing e-learning’s perceived usefulness. We were particularly concerned with which aspects might relate to how students perceive the usability of an online course’s e-content, and when an e-course helps them understand the learning content while studying for the final exam. According to Davis ([32], p. 320), perceived usefulness may be defined as: “The degree to which a person believes that using a particular system would enhance his or her job performance”. In models developed to explain the dynamics of technology adoption and use, namely, the Technology Acceptance Model (TAM) [23] and General Extended Technology Acceptance Model (GETAMEL) [33], the external factors that affect perceived usefulness are experience, subjective norms, enjoyment, computer anxiety and self-efficacy. The TAM model has been successfully used as a framework to study an e-learning system’s acceptance by students [34], also in the case of BL [29].

Perceived usefulness was also studied as a predictor of the intention to use information technology by Davis *et al*. [35] or as the measurement of e-learning systems’ success by Joo *et al*. [36], and Hsieh and Cho [37]. These studies show that perceived usefulness is a valid factor to measure and for predicting the effectiveness of e-learning systems. Perceived usefulness and perceived satisfaction both contribute to learners’ behavioural intention to use an e-learning system [38]. Consequently, identifying the factors impacting perceived usefulness can help support and enhance the effectiveness and efficiency of e-learning systems [15].

We mainly concentrated on perceived usefulness and the factors influencing it. An individual student’s opinion and preference regarding the subject matter and teacher’s engagement were also included as two more observed factors. Our students physically interact with the teacher and other students during face-to-face lectures on a weekly basis. Therefore, the research regarding an e-course’s perceived usefulness looked at the relationship between the face-to-face content delivery and online learning material, rather than social interactions or collaborative learning other studies revealed them to be important factors in BL [5,9,14,33,39,40].

Encompassing the quality, reliability and ease of use of an e-learning system, as well as technical support, the technology environment is an important factor when assessing online learning’s effectiveness. Easy access to the learning environment and system functionality are critical factors influencing use of the system. Still, a student holding greater digital skills is more confident in an ICT environment and this positively influences their self-efficacy when working with computer [5,8,12,15,26,27,29,38]. The above factors may be brought together within a single concept, which we name technology acceptance. Accordingly, the following hypothesis is proposed:

H1: Technology acceptance influences student attitudes to e-learning.

Manwaring *et al*. [41] reported that a student’s initial interest in the subject matter provokes positive feelings, enjoyment and interest in the activity (emotional engagement). Face-to-face lectures, whereby a teacher interestingly and attractively gives a talk, are more acceptable among students and regularly attended. Feldman [42] stated that teachers from various educational fields tend to be evaluated slightly differently due to difficulty of the subject matter, also indicating that some courses are more difficult to teach and, even, that certain subjects have better teachers than others. Therefore, students might accept and rate teachers (and subjects) differently due to possible variations in their attitudes, goals, motivation, learning styles, and perceptions of the teaching [43]. Casero [44] concluded that a student’s ratings of teaching effectiveness are correlated with the teacher’s personality as perceived by the student. The perceived usefulness of an e-course is unconsciously linked to the student’s affection for the teacher’s personality and the subject matter, as manifested in the satisfaction of both delivery methods, face-to-face and e-learning. Accordingly, two more hypotheses are posited:

H2: The perceived usefulness of an e-course depends on an individual student’s acceptance of the teacher’s personality.
H3: A student’s attitude to the teacher and the subject matter influences their preference for an e-course.

In e-learning, the course design, learning materials (a variety of activities etc.) and ease of using the e-course (simplicity of finding specific contents etc.) affect how a student experiences an e-course. It is reasonable to assume that some characteristics of an e-course positively impact a student’s perception of the e-learning environment.

A further significant aspect of e-learning is the teacher’s engagement in the online teaching environment [18,21,45]. The concept of the e-course’s design is independently edited by the teacher with the support of a staff member from the informatics department. The teacher sets the goals and provides the learning materials and activities, quizzes and assignments to achieve the goals. In recent research, Almerich *et al*. [24] investigated teachers’ ICT competencies and showed that teachers are crucial for integrating ICT into the educational process, meaning that teachers must master both technology and pedagogical knowledge. Responding to a student’s need or problem quickly and efficiently, giving online feedback about a student’s activities in an e-course such as assignments submitted or forum discussions, and posting relevant information about the course, are important factors for ensuring students are satisfied with an online learning environment [18,21,45]. The teacher must hence be capable of handling LMS, be active in synchronous (video conferences, chat) and asynchronous (forum posts, messages, feedback) communications and hold considerable skills for preparing relevant e-contents consistent with the face-to-face contents in an interesting and informative way, all of which we describe with use of the word “e-teaching”. On the other hand, Vo *et al*. [30] confirmed the moderating effect of subject discipline in blended learning. Some subject matters can take better advantage of the blending environment than others to facilitate the learning process. The following hypothesis is thus proposed:

H4: E-teaching influences the perceived usefulness of an e-course.

**Fig 1:**
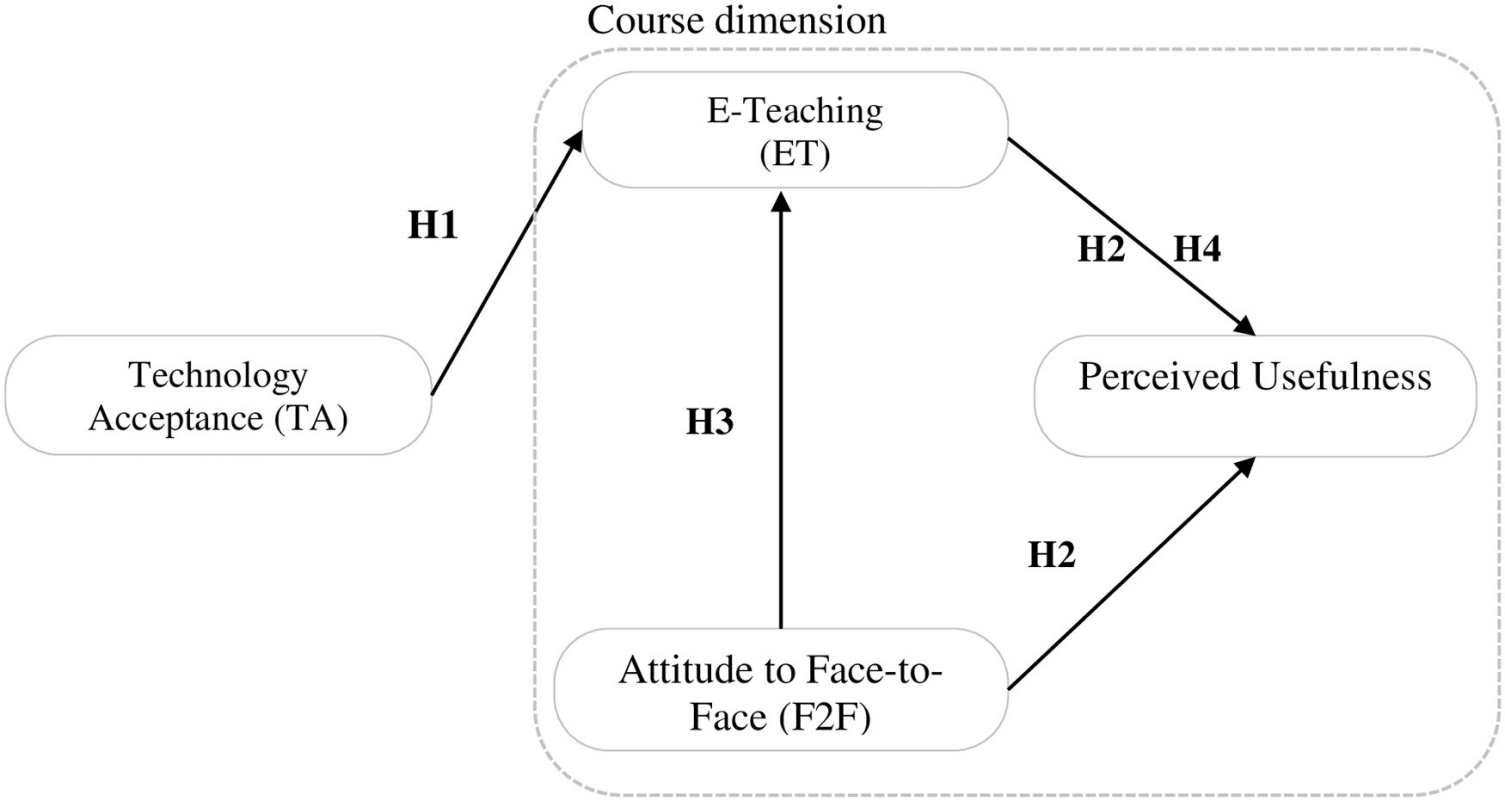
The proposed research model for student-perceived usefulness.

In summary, we propose the research model shown in Fig 1 and Table 1. The factor “*Technology Acceptance”* (TA) includes a student’s experience, computer confidence and self-efficacy, which are external factors of TAM and, in addition, the perceived ease of use. The factor *“Face-to-Face”* (F2F) combines a student’s affection for the subject and the teacher in a traditional way of teaching. On the other side, the third factor “*E-Teaching”* (ET) integrates aspects of an e-course as perceived by the student, including the teacher’s component of the design and organization of the e-course and if the teacher is assisting the student learning process. Relating to the challenges of ‘how to’ design BL [28], the factors F2F and ET including the learning climate of both methods and as well as the ET verified whether the teacher assists the students’ learning with timely feedbacks or comments. The fourth one is “*Perceived Usefulness*” (PU), the dependent variable, and measures students’ trust in the usability of an e-course with an impact on their learning achievement. Students’ perception of the usability of an e-course with an effect on their learning performance, i.e. students’ perceived usefulness of an e-course, reflects three aspects of the questionnaire (S1 Questionnaire):

EL4: The prepared learning material and tasks are consistent with the lectures in the classroom and supplement them.
EL5: The prepared material and assignments supplement the tutorials in the classroom.
EL6: Learning materials and activities in the e-course helped me to effectively study this subject matter.

**Table 1:** Factors and questionnaire items.

Factor	Questionnaire items (see S1 Questionnaire)
**Technology Acceptance (TA)**	
*Student factor*:	
computer confidence	CS1, CS5
attitude to the e-learning generally	CS6, CS7
*Technology factor*:	
ease of use	CS2
stable system	CS4
technical support	CS3
**Face-to-Face (F2F)**	
*Teacher factor*:	
way of teaching	FF3
*Student factor*:	
attitude to the subject matter	FF1, FF2
**E-Teaching (ET)**	
*Teacher factor*:	
structure and design of the e-course,	ET1, ET2, ET3, EL1, EL2, EL3
teacher’s engagement	ET4
*Student factor*	
attitude to the e-content	ET5, ET6
**Perceived Usefulness (PU)**	EL4, EL5, EL6

## Research method

### Survey instrument

Based on empirical findings from the literature [13,16,18,38,45], we developed a questionnaire to suit the BL provided at the FPA. The questionnaires are administered at the end of each semester in the Moodle environment.

The questionnaire measures 22 individual student attitudes to the e-learning environment of a given course: evaluation of the e-course layout (ET1–ET6) and impressions of the learning e-content (EL1–EL6), extended by three questions on their preference for face-to-face learning (FF1–FF3), and another seven general questions on digital skills, technical support and general attitudes to e-learning (CS1–CS7). All measures employ a seven-point Likert scale to obtain a greater variety of responses, ranging from 1 (“disagree very strongly”) to 7 (“agree very strongly”), with the additional option “I do not want to answer/no experience”. The questionnaire enabled us to investigate BL holistically over several courses from different years of study and various scientific disciplines (law, economics, informatics, and statistics) at the FPA.

In addition, students indicate their other regular weekly obligations apart from studying, e.g. student work, sports training, and cultural/artistic activity. Students are asked to insert their student ID number to enable the results obtained to be linked with certain demographic data from the student information system; gender, high school final grade, study programme, year of study and region of residence.

### Data

The data used in the study were taken from questionnaire-based surveys completed in two consecutive academic years, 2014/15 and 2015/16, by undergraduate FPA students. Since they were all invited to complete the survey, the participants in fact represent the entire population. We excluded 13 records where the answers to all questions had the same value (“straight-lining”), suggesting the responding student did not even read the questions. The merged dataset of e-courses contains 3,424 evaluations (records). An anomaly index based on all opinion questions was also computed. A visual inspection of the anomaly index’s distribution suggested that 27 records with an anomaly greater than 2.1 should be removed. In addition, 53 records with more than seven (one-half) of missing values were removed. In the pre-processed dataset, some values (<1%) were still missing. We replaced them with the default parameters used in the Expectation-Maximization (EM) algorithm in SPSS.

The final sample for the analysis included 3,334 records, each representing a student’s e-course evaluation; 539 students evaluated 46 e-courses; on average, each student evaluated 11.7 e-courses. Besides their regular weekly obligations, some demographic values from the students’ database were added: gender, high school final grade (graduation), year of study, programme, and region of residence (Table 2). Most respondents were female, which agrees with the fact most students at the FPA are female. The respondents’ average success at high school was 2.94 (max. 5). Most participating students (77.5%) had more than 2 hours of weekly obligations, such as sports participation, music education or student work–from 2 to 6 hours per week to more than 6 hours per day.

**Table 2:** Demographic profile of 539 respondents.

	Frequency	Percentage (%)
**Gender**		
Female	380	70.5
Male	159	29.5
**Regular weekly obligations**		
No activities or less than 2 hours per week	121	22.5
Less than 6 hours per week	118	21.8
More than the above	300	55.7
**High school final grade (graduation)**		
2 (fair)	203	37.7
3 (good)	210	39.0
4 (very good)	75	13.9
5 (excellent)	51	9.5
**Study programme**		
Professional	286	53.1
University	253	46.9
**Year of study**		
1st	319	59.2
2nd	143	26.5
3rd	77	14.3
**Region of residence**		
Ljubljana region	316	58.6
Other Slovenia	211	39.1
Abroad	12	2.2

### Statistical analysis

A structural equation model was used to answer the first research question: which factors influence an e-course’s perceived usefulness while learning for the final exam? The validity and internal reliability were checked using the SPSS statistical software and its AMOS extension. Before fitting the SEM model, descriptive statistics (means, standard deviation, Pearson’s correlations) was performed on the analysed variables. KMO and Bartlett’s test were used to test the feasibility of the component analysis, and reliability was tested using Cronbach’s alpha. Other measures of reliability (CR, AVE) and validity (SIC) were also tested. After fitting the SEM model, standard indicators of goodness of fit (RMSEA, NFI, CFI, TLI, NNFI, PNFI) were computed. A more detailed explanation of the statistical methods employed is found in the Results section. Further, the proposed model was tested on several subgroups to answer the second research question.

### Ethics statement

All students of the 1st-cycle programmes were invited to complete the survey voluntarily, without any coercion or undue influence. All respondents were over the age of 18. They were informed of the anonymity and confidentiality of the collected responses and research findings in the written introduction before completing the survey. A student’s identification number was only used to complement the responses with demographic data in the student database, and then the anonymization was done to prevent the study results being linked to any individual. According to standard socio-economic studies, no ethical concerns are involved other than preserving the participants’ anonymity. The authors confirm that this specific study was reviewed and approved by the Faculty Management and R&D Office before the study began. An official research ethics committee has not been established at the Faculty.

## Results

The tendency was to build a measurement model that fits the data while also satisfying the other validity and reliability indicators. In testing different combinations of manifest variables, 4 factors were established and 12 variables were included: TA with 2 items (CS2, CS4), ET with 4 items (ET1, ET2, ET3, ET4), F2F with 3 items (FF1, FF2, FF3) and PU, the dependent variable in the model, with 3 items (EL4, EL5, EL6). All the other manifest variables showed low factor loadings and were removed from the proposed research model.

### Descriptive statistics and measurement validation

The means and standard deviations for all four factors and corresponding items are shown in Table 3. The means indicate that most students tended to view e-learning positively since the mean scores all exceed 5 (on a scale from 1–7), ranging from 5.05 to 5.91.

**Table 3:** Descriptive statistics for factors and corresponding variables.

	Mean	Standard deviations
**TA**	5.700	1.201
CS2	5.810	1.251
CS4	5.590	1.150
**ET**	5.798	1.363
ET1	5.770	1.308
ET2	5.890	1.306
ET3	5.910	1.248
ET4	5.620	1.588
**F2F**	5.150	1.628
FF1	5.080	1.566
FF2	5.050	1.677
FF3	5.320	1.640
**PU**	5.617	1.393
EL4	5.710	1.327
EL5	5.680	1.330
EL6	5.460	1.522

To identify the correlation between the items, a bivariate Pearson correlation was calculated. Due to the large sample size (n = 3,424), all pairs of analysed variables were significantly correlated at the level of 0.01 (2-tailed). Higher correlations (>0.5) were exposed (Table 4). The variables in the ET factor had the strongest correlation with the PU variables. On the other side, TA showed a lower yet a still significant correlation with them.

**Table 4:** Correlations between the variables (correlation is significant at the 0.01 level (2-tailed)).

	**CS2**	**CS4**	**FF1**	**FF2**	**FF3**	**ET1**	**ET2**	**ET3**	**ET4**	**EL4**	**EL5**	**EL6**
**CS2**	1											
**CS4**	0.496	1										
**FF1**	0.106	0.161	1									
**FF2**	0.076	0.136	**0.769**	1								
**FF3**	0.116	0.156	**0.600**	**0.537**	1							
**ET1**	0.289	0.290	0.374	0.368	0.434	1						
**ET2**	0.225	0.261	0.320	0.319	0.400	**0.682**	1					
**ET3**	0.203	0.240	0.349	0.350	0.390	**0.594**	**0.624**	1				
**ET4**	0.109	0.187	0.175	0.176	0.329	0.414	0.465	0.374	1			
**EL4**	0.193	0.235	0.372	0.414	0.404	**0.513**	**0.516**	**0.522**	0.375	1		
**EL5**	0.184	0.227	0.359	0.388	0.440	**0.512**	**0.514**	**0.511**	0.376	**0.766**	1	
**EL6**	0.176	0.208	0.374	0.370	0.447	**0.502**	0.462	0.464	0.349	**0.573**	**0.611**	1

KMO and Bartlett’s test revealed the KMO measure of sampling adequacy was 0.869 and the p-value for Bartlett’s test was <0.0001. This shows the component analysis was feasible. Principal component analysis was thus employed to test the factor validity (Table 5). Four factors were extracted and were consistent with the hypothesized construct. All eigenvalues were not greater than 1 according to the Kaiser criterion, i.e. the eigenvalues-greater-than-1 rule [46], although the rule is the result of misapplication of a formula for internal consistency reliability and would under-estimate the number of factors [47].

**Table 5:** Total variance explained (extraction method: Principal component analysis).

Factor	Extraction sums of squared loadings			Rotation sums of squared loadings		
	Total	% of Variance	Cumulative %	Total	% of Variance	Cumulative %
ET	5.238	43.651	43.651	2.553	21.274	21.274
F2F	1.441	12.011	55.663	2.362	19.687	40.961
PU	1.268	10.570	66.233	2.279	18.995	59.956
TA	0.789	6.577	72.809	1.542	12.853	72.809

According to MacCallum *et al*. [48], it is appropriate to perform factor analysis if the proportion of the total variance explained (i.e. the average of all communalities) exceeds 0.7. In our case, the average communality was 0.728, implying that our questionnaire held high construct validity (according to the data).

Indicators of the model factors’ reliability and validity were calculated (Table 6). The reliability of each factor exceeded the 0.7 threshold, except for TA. As seen in Table 3, the TA factor consisting of only 2 items seems to be more problematic but, since it consists of a small number of items, it is appropriate to consider a cut-off value of 0.6 [49]. Therefore, the measurement scales were valid and reliable and composite reliability (CR) was achieved. Convergent validity was achieved for all four factors. Internal consistency was identified using Cronbach’s alpha coefficient.

**Table 6:** Reliability statistics.

Factor	Item	Cronbach’s alpha	CR	AVE
TA	2	**0.661**	0.772	0.500
ET	4	0.805	0.888	0.547
F2F	3	0.838	0.910	0.659
PU	3	0.843	0.914	0.667

Confirmatory factor analysis was used to test whether the model adequately fits the data. To achieve discriminant validity (SIC), the square root of AVE for each factor must be greater than the inter-factor correlations. As Table 7 shows, the diagonal elements of the matrix exceeded the off-diagonal elements, with one exception–the correlation between ET and PU (0.573), which slightly exceeded the corresponding AVE (0.547). Even so, discriminant validity is shown when each measurement item correlates weakly with all the other factors, except for the one with which it is theoretically associated [50]. Therefore, all factors in the model displayed satisfactory reliability and discriminant validity. All factor loadings were higher than 0.5, indicating that all the latent variables were well represented by the indicators given the sample size [51].

**Table 7:** Discriminant validity (SIC). The numbers in the diagonal row are square roots of AVE.

	TA	ET	F2F	PU
TA	0.500			
ET	0.197	0.547		
F2F	0.060	0	0.659	
PU	0.123	**0.573**	0.299	0.667

### Structural model examination

**Fig 2:**
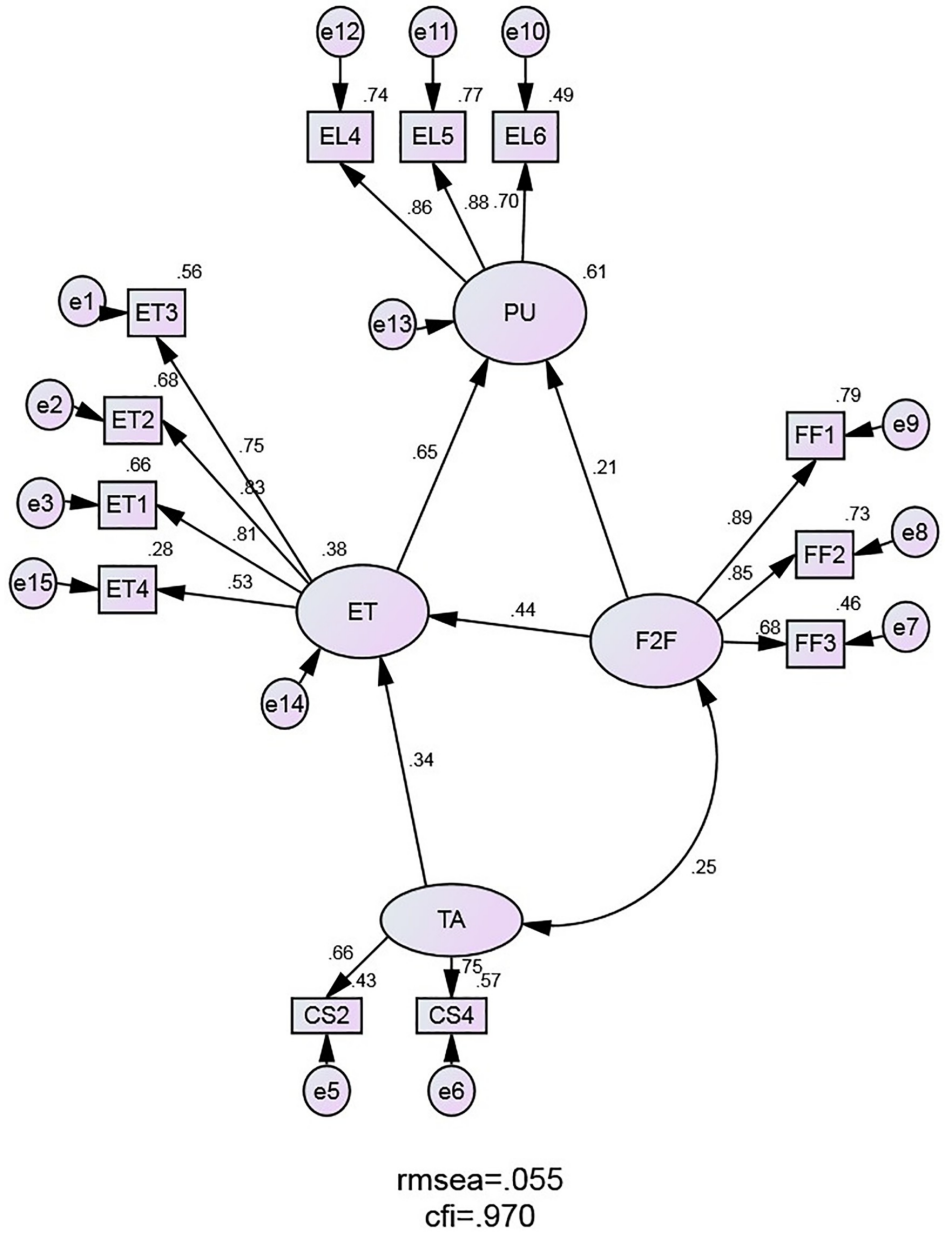
The structural equation model (standardized values are shown).

Based on the proposed research model and statistical analysis described in the previous section, the final structural model was confirmed (Fig 2). All specified paths between the model factors had significant coefficients. The factors TA, ET and F2F explained 61% of the variance of PU. TA and F2F were exogenous factors in the model, with F2F having a direct effect on PU and TA an indirect effect on PU. The strongest effect was recorded with ET on PU (0.65), the weakest F2F on PU (0.21).

The fit indices reveal the structural model fits the data satisfactorily (Table 8). The absolute and incremental indices exceed the recommended values; the model is parsimonious. CMIN (with df and p) is not reliable given the large sample size and RMSEA indicates the good fit of the model [48].

**Table 8:** Indicators for measuring the model’s adequacy.

Indicator		Recommended value	Value
Minimum of discrepancy (χ2)	CMIN		540.329
Degrees of freedom	df		49
p value	p	>0.05	<0.001 0.000
Root mean square error of approximation	RMSEA	<0.05 (0.01, 0.05 and 0.08 indicate an excellent, good and mediocre fit)	0.055
Bentler-Bonett index/Normed fit index	NFI	>0.90	0.967
Comparative fit index	CFI	>0.90	0.970
Tucker Lewis index/Non-normed fit index	TLI NNFI	>0.90	0.952
Parsimonious normed fit index	PNFI	>0.60	0.608

## Discussion

The students’ opinions on different aspects of BL were measured using a questionnaire, aspects such as: (1) the characteristics of an e-course (goals, materials and assignments); (2) teachers’ activities and support (assessments, responses, feedback); (3) students’ preferences for learning online versus traditional face-to-face learning; and (4) the ICT aspect (computer confidence, stability, administrative support). In the empirical analysis that explored the relationships between some aspects of BL, the largest share of excluded variables was found in the factor TA. It may be expected that skills such as working with computers (CS1, CS5) are very common among young people today. Further, the reliability and stability of the system (CS3) is also excluded, probably because LMS Moodle can be found all over the world at different levels of education and is continuously upgraded. However, ease of using the system (CS2) and technical support are very important for the students (CS4). The last two aspects about e-learning’s contributions to learning success and replacing F2F teaching (CS6, CS7) are more purpose-oriented and do not focus on the TA. Therefore, they do not load on the same factor.

According to previous literature [13,19,45], aspects influencing a student’s satisfaction with e-learning are the design of an e-course, such as clarity of the structure (ET1), ease of use (CS2), and the teacher’s administration of it (knowledge and use of the LMS functions). Offering a variety of assessments (ET3), i.e. quizzes, written work, forums etc., is in harmony with the finding that several shorter assignments in an e-course positively affect a student’s outcome [30]. Moreover, timely feedback is even more important (ET4) [22,31]. In our model–except for ease of use, which is part of the technology factor–the other aspects were elements of the ET factor. In addition to the mentioned aspects, another important variable in e-teaching was revealed, namely the clarity of the stated goals at the beginning of the semester (ET2). The goals that are set allow the student to plan and organize their activities during the semester, which is particularly helpful for a student who is not self-organized.

The results demonstrate the perceived usefulness was positively influenced by all three factors, as proposed in the research model. The ET and F2F factors had a direct impact, while TA had an indirect impact. The structural model confirmed the modest relationship between TA and ET. More precisely, we corroborated that the simplicity of use of e-courses and satisfaction with the technical support given positively influence students’ acceptance of e-learning. In particular, they increase their perception of an e-course’s organization, the clarity of the workload demands, the variety of activities, and the teacher’s feedback. The empirical findings are consistent with previous research [14,27]. Second, we showed the e-teacher’s aspect has a stronger impact on the perceived usefulness of an e-course than face-to-face learning. This agrees with previous research studies that reveal course design is an aspect that influences performance, satisfaction and dropouts in an e-course [10,13,14,41,45]. At the same time, the face-to-face aspect influences the assessment of e-teaching. This may be attributed to the fact that, when the teacher is personally ‘attractive’ and the course topics are of interest to a student, the student more easily overlooks smaller deficiencies of an e-course.

We also confirmed our third hypothesis that sought to link the dimensions of face-to-face learning with e-teaching; namely, that attitudes to a subject influence a student’s preference for an e-course. Our last claim that e-teaching influences the perceived usefulness of an e-course and the attitudes to the e-course combines our second and third research hypotheses, which were both confirmed.

### Multiple subgroup model testing

Due to the considerable variety of the students involved in the research, conclusions for the whole dataset may not hold for specific student subgroups. That is, our previous work revealed some differences among students at the FPA based on demographic characteristics [52]. Therefore, the proposed model was tested on several subgroups. It turned out that the model also applies to the subgroups, with just a few factors affecting the different groups.

Although gender differences in learning style are confirmed [53,54], and recent research again shows that males hold more favourable attitudes to ICT than females, albeit they are still positive [55], testing the structural model on gender revealed no such difference. In the model, the variance explained for PU showed that ET, TA and F2F explained greater PU variance in the case of male (69%) than female (58%) students, which is not surprising because there are significantly more females in the student population. The effect of ET on PU was slightly greater for males (females 0.64, males 0.69). The covariance between F2F and TA was greater for females (0.29>0.17). Two indicators for measuring the model’s adequacy, RMSEA and CFI, were the same for both gender subgroups (RMSEA = 0.039, CFI = 0.970). Therefore, this fact allows the model to be generalized to both males and females.

In the case of study programme–university or professional–almost all model parameters were practically the same, except for the F2F on ET effect which had a bigger impact on the professional study programme segment (0.51>0.37). A possible explanation for this finding is that university-programme students are better at self-organizing. Therefore, the role of face-to-face learning in increasing attitudes to ET is not so important.

The following differences between levels of education were found. For 1st-year students, the model’s variance explained of PU was 0.59, for the 2nd-year students it was 0.66, and for the 3rd-year students 0.57. Segmentation produced various F2F on PU effects (1st year 0.20, 2nd year 0.15, 3rd year 0.27). The F2F on PU effect was greatest for 3rd-year students and lowest for 2nd-year students. The ET on PU effect (1st year 0.65, 2nd year 0.72, 3rd year 0.59) was strongest among 2nd-year students, while MT on ET (1st year 0.37, 2nd year 0.28, 3rd year 0.30) was slightly greater for the 1st-year students. The influence of F2F on ET (1st year 0.46, 2nd year 0.44, 3rd year 0.40) had almost the same effect regardless of the year of study. Covariance between F2F and AT (1st year 0.17, 2nd year 0.37, 3rd year 0.29) was greatest among the 2nd-year students. Still, RMSEA (0.035) and CFI (0.964) were the same for all three subgroups.

In the case of students’ weekly obligations, the generalization of the model for all segments was reliable (RMSEA = 0.036, CFI = 0.961), while the highest share of variance explained for PU was in the second group in which students work (besides the study) less than 6 hours a week (0.65). F2F on ET has the strongest impact in that segment (0.52). Further, the F2F on ET effect was weakest in the segment where students work more than 6 hours per week (0.38). This means: the more they work, the less important is the F2F on ET effect. However, in this segment the ET on PU effect was the strongest (0.67). One explanation for this may be that students with plenty of activities apart from study do not attend the lectures and tutorials so regularly. Therefore, the e-courses play an important role as a source of relevant information for them when learning for final exams.

## Conclusion

Understanding the factors that affect how students perceive an e-course’s usefulness may be of considerable value for teachers and e-learning management when they are seeking to design an e-course that is more useful and helpful for students preparing for the final exam. Based on a questionnaire-based survey, the impacts of some factors on perceived usefulness in the higher education environment were investigated. BL requires complex teaching strategies where, in addition to supporting face-to-face lectures, students’ contributions and other activities in an e-course require continuous monitoring. To successfully employ BL in higher education, it is important to know how well e-learning supports the face-to-face lectures, if the quality of the e-content is suitable, or whether an e-course has been designed for its own sake.

Our research confirmed the importance of learning climate and social interaction, mainly the teacher–student interaction in a BL course [28]. Namely, e-teaching shows a positive influence on an e-course’s perceived usefulness. Students hold positive views of various types of activities in an e-course, individual help, and the teacher’s feedback. If the course material and activities are well prepared and easy to use, students feel comfortable and have greater motivation to study. A positive learning climate depends on the efficient interaction of the student and the teacher, especially by way of quick feedback and frequent interaction in the e-course. Although in BL students also have weekly contact with the teacher in the classroom, it is important they contact the teacher through the messages or forums in an e-course. They also expect timely feedback, which motivates them to learn [14]. This fact suggests the teacher should be confident in the e-learning environment. Further, teachers should be competent in both pedagogical and technological fields to introduce various ICT resources into their teaching process and, interestingly, technological competencies influence pedagogical ones such that technological competencies provide the basis for pedagogical ones [24]. Therefore, the FPA should provide guidelines for the teaching-learning process with ICT and enable the professional development of the teachers to help them become independent, efficient and confident in using the various functionalities of the e-learning system. The informatics staff responsible for the LMS should inform teachers of the system’s novelties and be available to help them whenever they need it.

As technology aspects positively influence e-teaching, management of the faculty should provide an introductory course for 1st-year students with insufficient skills and knowledge to use computers and, finally, a seminar on how to use the LMS tools. This would equip students with the skills they need to become confident and satisfied in the e-learning environment and encourage them to more actively participate in an e-course.

The study has some limitations that may be the subject of future research. First, the student survey used in this study was not mandatory. The sample could be biased, i.e. students with different attitudes to BL may have had different intentions to participate in the survey. In addition, there was a large drop in the number of questionnaires completed by students in the 2nd and 3rd years of study. To capture the most representative sample in future surveys, we will try to motivate as many students as possible to complete the survey. The main task will be to convince the students that the results obtained are useful for them and that their participation in the survey is beneficial.

Observed aspects of the e-course were only measured with a questionnaire. This is an individual judgement of a student which could be upgraded with an objective dimension of a student’s activities in an e-course acquired from the LMS student log file. Adding the correlation between perceived usefulness and the success of students in the final exam could give a better insight into the effectiveness of e-learning. Moreover, a questionnaire for teachers evaluating activities in an e-course could be created to measure which activity they consider is the most helpful for the students. The results from both sides could be aggregated to the level of an e-course to identify any overlap in their opinions.

Other research methods could be applied to find interesting patterns in the data. Use of the Orange software [56] might allow interesting subgroups of students to be characterized using different statistical methods (clustering, statistical classification, subgroup discovery, multidimensional visualizations, network analysis etc.).

Finally, our research was carried out in a specific higher education environment (that deals with the study of public administration) that uses a particular LMS. Thus, any generalizations to other e-learning environments must be made with care.

## Supporting information

S1 Questionnaire Questionnaire items used in the research. (DOCX)

S1 Data Processed data. (CSV)
