# Supplementary material for: Exploring critical factors of the perceived usefulness of blended learning for higher education students
Source: PLoS One. 2019 Nov 21;14(11):e0223767. doi: 10.1371/journal.pone.0223767 (PMC6872162; doi:10.1371/journal.pone.0223767)
Supplement: S1 Questionnaire — (DOCX) [file pone.0223767.s001.docx]

| **Label** | **Questionnaire items** |
| --- | --- |
| Computer Self-efficacy & System Functionality | |
| CS1 | Working with computers for study purposes suits me. |
| CS2 | The Moodle e-learning system is easy to use. |
| CS3 | The Moodle system is reliable and stable (it does not crash, submitted tasks are not lost). |
| CS4 | I am satisfied with the support and assistance in the event of technical problems. |
| CS5 | Working with computers for study purposes is not difficult for me. |
| CS6 | E-learning contributes to higher student academic performance. |
| CS7 | E-learning is a quality replacement for traditional learning in the classroom. |
| Course dimension – F2F | |
| FF1 | The content of the course interests me. |
| FF2 | Course lectures are interesting for me and I like to attend them. |
| FF3 | I find the face-to-face tutorial attractive and useful. |
| Course dimension – online teaching (teacher dimension) | |
| ET1 | The e-classroom of the course is organized transparently. |
| ET2 | The goals (workload demands, grading) of this e-course were clearly stated at the beginning of the semester. |
| ET3 | This e-course offers a variety of ways of assessing my learning (quizzes, written work, forums, files…). |
| ET4 | I receive the teacher's comment/feedback on an assignment within less than 7 days. |
| ET5 | I prefer fewer lectures in the traditional way (face-to-face) and more learning material processed in the e-course. |
| ET6 | More course exercises could be carried out in the e-course instead of in the classroom. |
| Course dimension – online learning (learner dimension) | |
| EL1 | The general impression of the e-course is good. |
| EL2 | Study material and tasks of the e-course are presented in a clear and understandable way. |
| EL3 | Finding certain activities in the e-course is simple. |
| EL4 | The prepared learning material and tasks are consistent with the lectures in the classroom and supplement them. |
| EL5 | The prepared material and assignments supplement the tutorial in the classroom. |
| EL6 | Learning materials and activities in the e-course helped me to effectively study this subject matter. |
